# Supplementary material for: scAmpi—A versatile pipeline for single-cell RNA-seq analysis from basics to clinics
Source: PLoS Comput Biol. 2022 Jun 3;18(6):e1010097. doi: 10.1371/journal.pcbi.1010097 (PMC9200350; doi:10.1371/journal.pcbi.1010097)
Supplement: S1 Text — (DOCX) [file pcbi.1010097.s001.docx]

**SUPPLEMENTS for manuscript “scAmpi - A versatile pipeline for single-cell RNA-seq analysis from basics to clinics”**

**S1 Text: Parameter setting and analysis call**

Basic scRNA:

snakemake -s snake_scAmpi_basic_master.snake --configfile config_MelanomaSample.json

Clinical:

snakemake -s snake_scAmpi_clinical_master.snake --configfile config_MelanomaSample.json

Analysis-specific parameters and resources required by the pipeline can be provided in a configuration file in json format. An example file listing the default settings is provided on the git repository.

Specific parameters for the melanoma showcase example:

Normalization:

MT-fraction threshold = 0.5

Number of genes = 400

Clustering:

Number of neighbors = 30

Minimum number of cells per cluster = 20

Number of highly variable genes = 2000

Clinical part:

clinicaltrials.gov key words: “solid tumor, melanoma”
