# Supplementary material for: scAmpi—A versatile pipeline for single-cell RNA-seq analysis from basics to clinics
Source: PLoS Comput Biol. 2022 Jun 3;18(6):e1010097. doi: 10.1371/journal.pcbi.1010097 (PMC9200350; doi:10.1371/journal.pcbi.1010097)
Supplement: S2 Text — (DOCX) [file pcbi.1010097.s002.docx]

**SUPPLEMENTS for manuscript “scAmpi - A versatile pipeline for single-cell RNA-seq analysis from basics to clinics”**

**S2 Text: Cell types and immune gene expression**

**
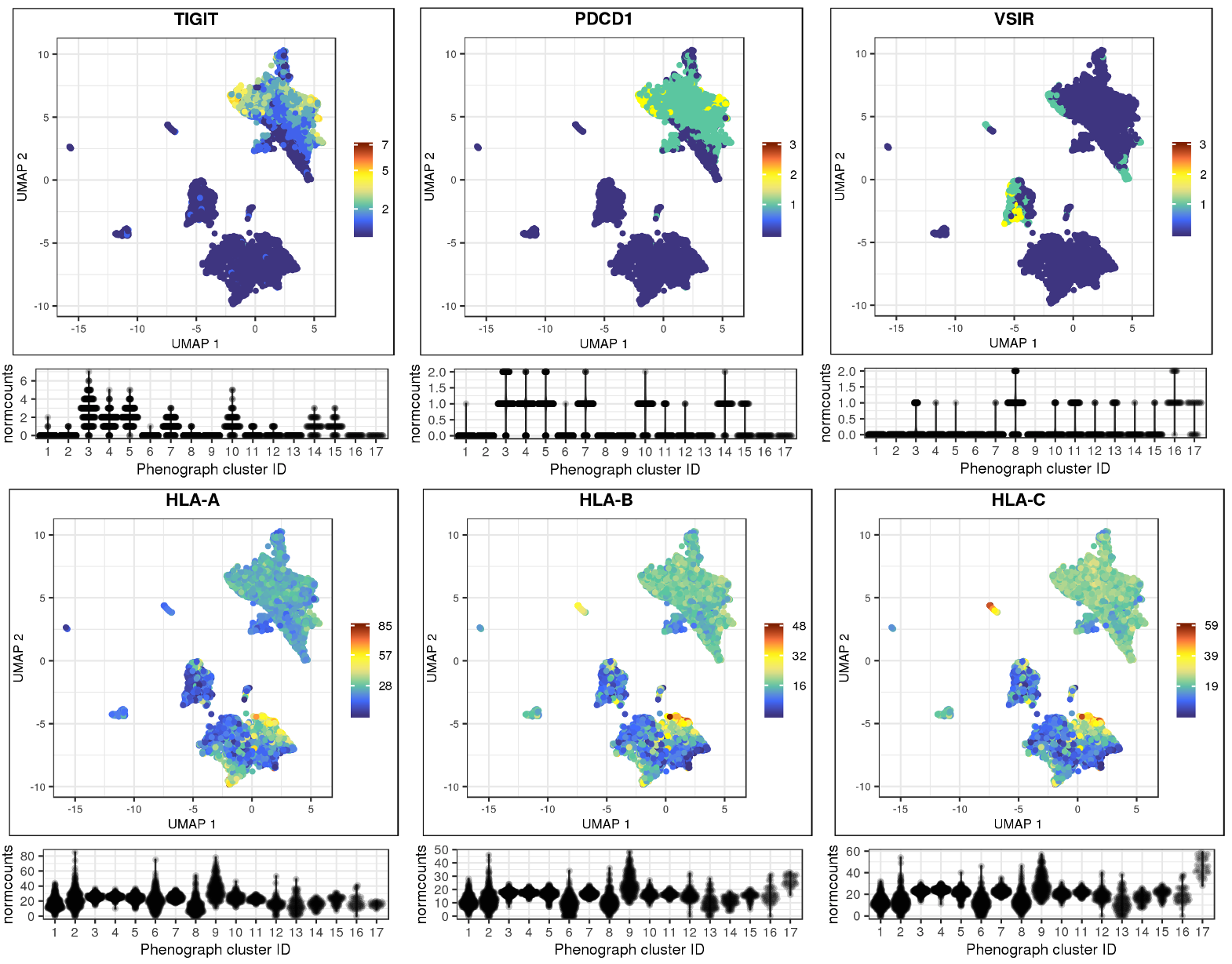
**

*Fig A: Single cell UMAPs and violin plots showing the normalized expression counts of immunotherapy-relevant marker genes (gene to protein names: PDCD1 = PD-1; VSIR = VISTA).*

*
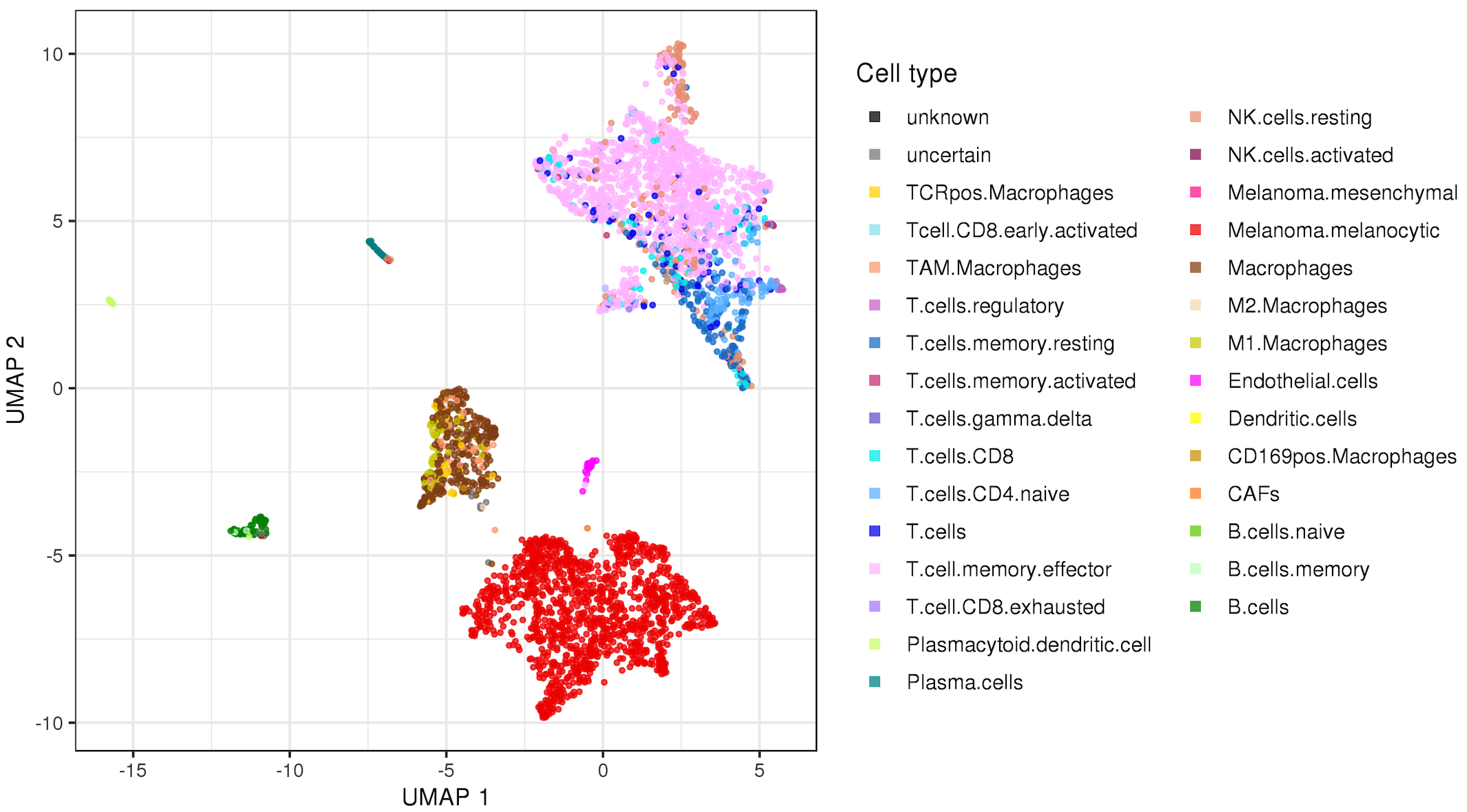
Fig B: Complete overview of the cell type composition of a melanoma biopsy sample.*
