# Supplementary material for: scAmpi—A versatile pipeline for single-cell RNA-seq analysis from basics to clinics
Source: PLoS Comput Biol. 2022 Jun 3;18(6):e1010097. doi: 10.1371/journal.pcbi.1010097 (PMC9200350; doi:10.1371/journal.pcbi.1010097)
Supplement: S3 Text — (DOCX) [file pcbi.1010097.s003.docx]

**SUPPLEMENTS for manuscript “scAmpi - A versatile pipeline for single-cell RNA-seq analysis from basics to clinics”**

**S3 Text: Tumor heterogeneity**

Figs A and B show selected examples of the gene set enrichment analysis based on differentially expressed genes and GSVA, respectively. The heat map illustrates that e.g. Interferon alpha/gamma response are up-regulated in cluster 9 but down-regulated in cluster 6. In combination with the GSVA score-colored UMAP it becomes apparent that the tumor is generally down-regulated for interferon alpha/gamma, with the exception of cluster 9.

*
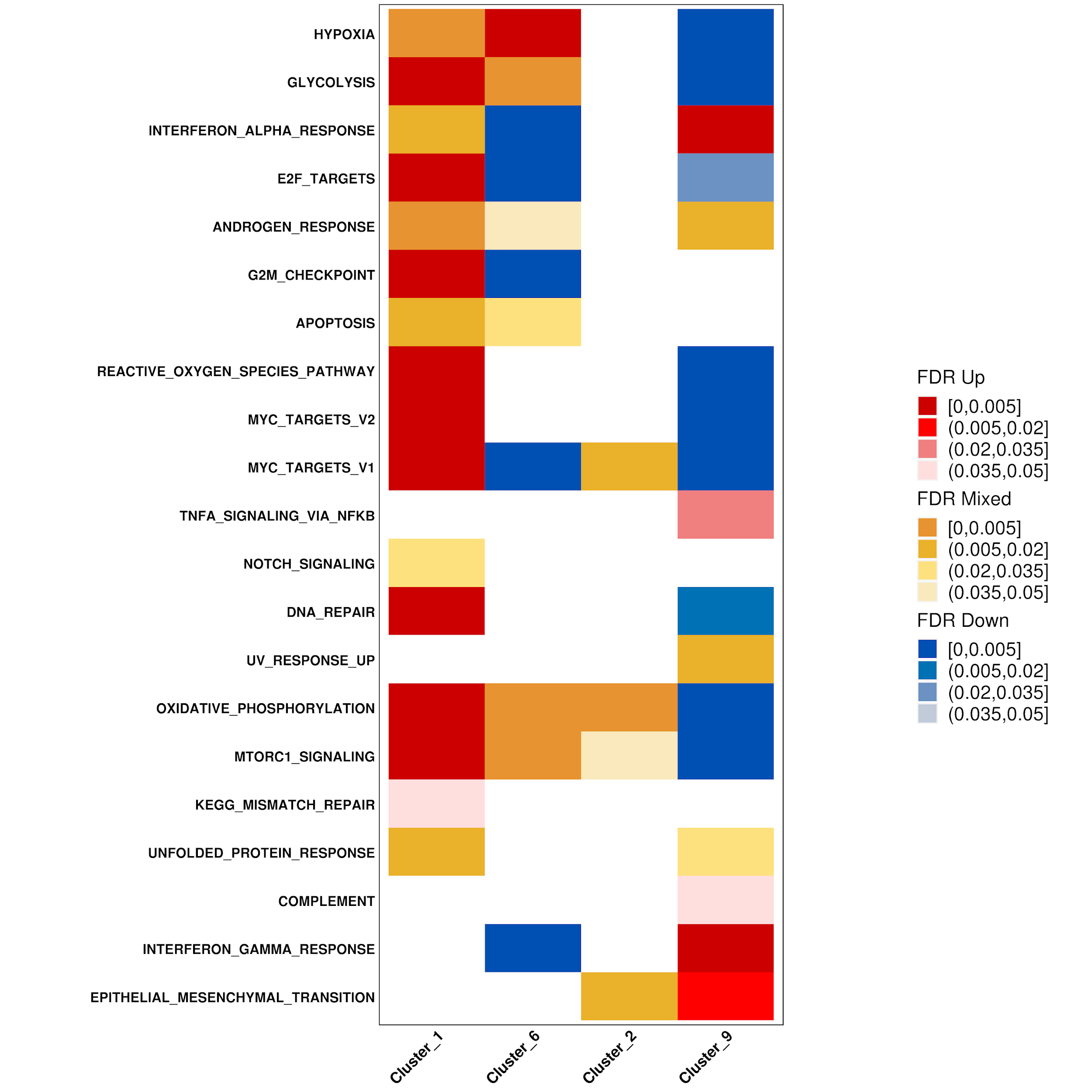
 Fig A: Heat map illustrating the gene set enrichment analysis results based on genes differentially expressed comparing malignant clusters with each other.*


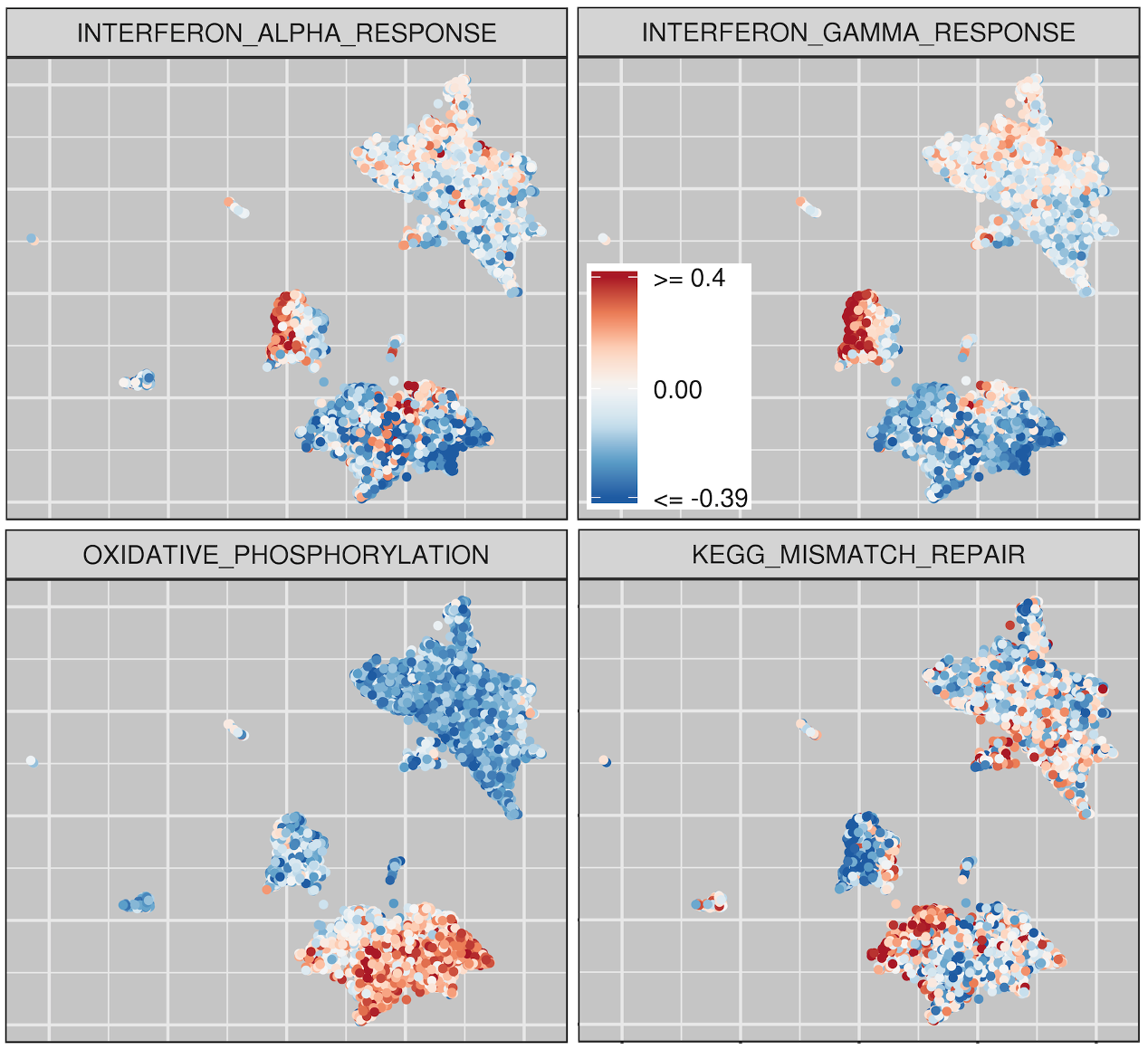


*Fig B: UMAPs that illustrate selected examples of the GSVA based gene set enrichment analysis.*
